# Identifying multimodal signatures underlying the somatic comorbidity of psychosis: the COMMITMENT roadmap

**DOI:** 10.1038/s41380-020-00915-z

**Published:** 2020-10-15

**Authors:** Emanuel Schwarz, Dag Alnæs, Ole A. Andreassen, Han Cao, Junfang Chen, Franziska Degenhardt, Daria Doncevic, Dominic Dwyer, Roland Eils, Jeanette Erdmann, Carl Herrmann, Martin Hofmann-Apitius, Tobias Kaufmann, Nikolaos Koutsouleris, Alpha T. Kodamullil, Adyasha Khuntia, Sören Mucha, Markus M. Nöthen, Riya Paul, Mads L. Pedersen, Andres Quintero, Heribert Schunkert, Ashwini Sharma, Heike Tost, Lars T. Westlye, Youcheng Zhang, Andreas Meyer-Lindenberg

**Affiliations:** 1 Department of Psychiatry and Psychotherapy, Central Institute of Mental Health, Medical Faculty Mannheim, Heidelberg University, Mannheim, Germany; 2 Norwegian Centre for Mental Disorders Research (NORMENT), Division of Mental Health and Addiction, Oslo University Hospital and Institute of Clinical Medicine, University of Oslo, Oslo, Norway; 3 Institute of Human Genetics, University of Bonn, School of Medicine & University Hospital Bonn, Bonn, Germany; 4 Department of Child and Adolescent Psychiatry, Psychosomatics and Psychotherapy, University Hospital Essen, University of Duisburg-Essen, Duisburg, Germany; 5 Health Data Science Unit, Heidelberg University Hospital and BioQuant, Heidelberg, 69120 Germany; 6 Department of Psychiatry and Psychotherapy, Section for Neurodiagnostic Applications, Ludwig-Maximilian University, Munich, 80638 Germany; 7 Center for Digital Health, Berlin Institute of Health and Charité, Berlin, 10117 Germany; 8 Institute for Cardiogenetics, University of Lübeck, DZHK (German Research Centre for Cardiovascular Research), partner site Hamburg/Lübeck/Kiel, and University Heart Center Lübeck, Lübeck, Germany; 9 Fraunhofer Institute for Algorithms and Scientific Computing (SCAI), Sankt Augustin, 53754 Germany; 10 Max-Planck Institute of Psychiatry, Munich, Germany; 11 Department of Genomics, Life & Brain Center, University of Bonn, Bonn, Germany; 12 Department of Psychology, University of Oslo, Oslo, Norway; 13 Department of Cardiology, Deutsches Herzzentrum München, Technische Universität München, Munich Heart Alliance (DZHK), Munich, Germany

**Keywords:** Diagnostic markers, Genetics

Schizophrenia carries an excess mortality of over 10 years that is largely due to somatic comorbidity with disorders including type 2 diabetes and cardiovascular diseases (CVD) [1, 2]. With metabolic abnormalities found in medication-naïve and first-episode patients, and an increased type 2 diabetes risk in relatives of individuals with schizophrenia, there is substantial evidence that the elevated prevalence of type 2 diabetes in schizophrenia is a partially treatment-independent comorbidity [3]. Individuals with schizophrenia also show a 50% increased risk of dying from CVD compared to the general population, which accounts for even more premature deaths [4]. Besides an unhealthy lifestyle, the CVD incidence is attributed to risk factors believed to be comorbid with schizophrenia, and genetics studies suggest an underlying pleiotropy that supports shared mechanistic effects between both conditions [5]. As patients are treated largely by a “one-fits-all” approach, there is a strong need to identify biological means to stratify patients and characterize the biological basis of somatic comorbidity. This will allow improved clinical delineation of psychoses and facilitate novel intervention strategies targeted at the minimization of comorbidity risk, reducing mortality and morbidity.

The multidisciplinary COMorbidity Modeling via Integrative Transfer machine learning in MENTal illness (COMMITMENT, https://www.sys-med.de/en/) project aims to address the above challenges. COMMITMENT leverages extensive multi-OMICs and neuroimaging data, which have recently provided an unprecedented opportunity for devising systems medicine approaches to psychiatric disorders that have lagged behind those successfully used for somatic illnesses. Adjusting such approaches to psychiatry and building on bioinformatics environments that can optimally fuse large-scale data that is physically distributed and cannot be easily merged may help us further understand the disorders’ underlying biology. To address this, COMMITMENT will build a transdiagnostic, translational research framework focused on disentangling the biological heterogeneity underlying psychoses and the identification of mechanisms shared with common somatic comorbidities.

**Fig. 1:**
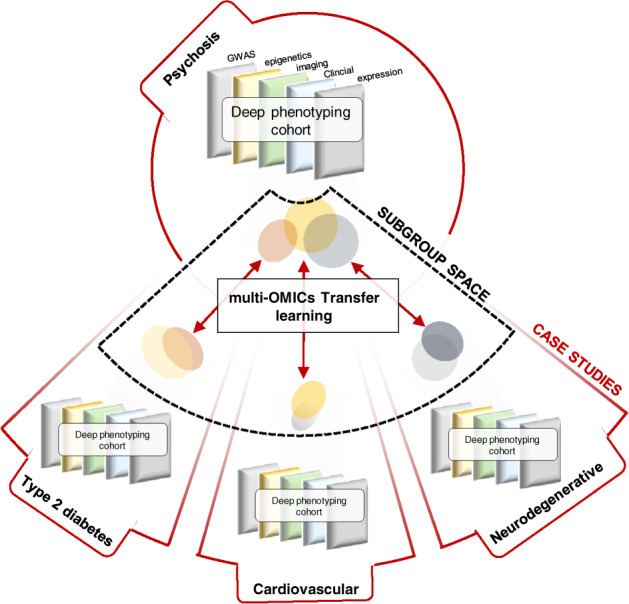
Schematic overview of the transfer-learning procedure. Biological signatures are transferred across cohorts, to identify biological dimensions that are simultaneously associated with psychosis and somatic comorbidities. Notably, the transfer of illness-signatures across cohorts allows circumventing the requirement of data from patients affected simultaneously by psychosis and comorbid conditions. Rather, subgroup profiles of these conditions are derived independently from disease-specific data and optimized for multivariate overlap in the feature space. COMMITMENT will build on these recent advances to develop a distributed and privacy-preserving computational framework that obviates the need for exchanging raw data across project partners, allowing analysis of the largest possible sample sizes while maintaining data security.

A core element of the COMMITMENT project will be the development of a computational pipeline that builds on the so-called “transfer learning”, which allows the “transfer” of signatures across cohorts [6, 7], i.e., the identification of patients diagnosed with psychosis along a biological dimension that also indexes patients with, e.g., type 2 diabetes (Fig. 1). Two further core components of the COMMITMENT approach are as follows: (I) the integration of prior information on biological mechanisms into the machine-learning approach and (II) the adaptation of computational approaches successfully used in the oncological field to optimally use multi-OMICs data for illness signature identification. First, the integration of mechanistic knowledge into machine learning models is aimed at improving our ability to identify biologically relevant signatures from multimodal data through a meaningful reduction of the data dimensionality. For this, a priori knowledge about mechanistic causes-and-effects linked to psychiatric conditions and comorbidities will be extracted at large-scale from the scientific literature, and made amenable for data mining. Second, machine learning approaches will utilize a nonnegative matrix factorization strategy, which allows integrative learning across data modalities [8]. COMMITMENT aims to extend this approach to incorporate the ability to project non-overlapping multimodal data into a smaller shared feature subspace (e.g., a shared pathway-level subspace). This will facilitate the integrative analysis of multimodal datasets where individual modalities are missing, and thus maximize the utilization of the available data resources.

In parallel, the COMMITMENT data resource will be expanded by predicting the genetically regulated component of expression data using PrediXcan/MetaXcan [9]. The transcriptome-wide association study will be performed for a wide range of tissues and cell types (obtained, e.g., from the Genotype-Tissue Expression, GTEx [10]), in order to identify specific cell types in which the causal genes exert their effects underlying psychosis and somatic comorbidities.

Evidence has also pointed toward an apparent brain aging in patients with mental disorders suggesting the presence of neurodegenerative processes. Their clinical relevance has yet to be characterized. Therefore, COMMITMENT will incorporate a developmental perspective on the identification of psychosis subgroups and the emergence of comorbid somatic phenotypes. In a first step, we will provide a detailed characterization of the structure of psychiatric symptoms, based on a dimensional representation of symptoms. This will allow exploring whether comorbidity-defining biological profiles map to clinically distinct, potentially transdiagnostic symptom profiles. We will then generate lifespan trajectories of somatic comorbidity profiles that can be tested for interactions with identified signatures indexing psychosis susceptibility, to identify age-periods with high comorbidity risk, and to disentangle state- vs. trait-related effects.

Finally, COMMITMENT will validate algorithms regarding their ability to stratify patients with psychotic symptoms, predict differential treatment response and outcome, as well as early signs of comorbidity onset.

In summary, COMMITMENT is a large, multidisciplinary effort to identify clinically relevant, multimodal signatures underlying different dimensions of psychosis and common somatic comorbidity. We are actively seeking for feedback on data analytics approaches as well as collaborations. With this, COMMITMENT will provide the basis for biologically informed clinical tools for improved personalized care of patients with psychotic symptoms in the hope of reducing the substantial excess mortality of this condition.
